# Iatrogenic botulism after intragastric botulinum neurotoxin injections – a major outbreak

**DOI:** 10.1186/s42466-024-00350-3

**Published:** 2024-10-29

**Authors:** Tsepo Goerttler, Martin B. Dorner, Christina van der Linden, Ricardo Kienitz, Stephan Petrik, Stephan Blechinger, Jonah Spickschen, Iris R. Betz, Carl Hinrichs, David Steindl, Frederike Weber, Thomas Musacchio, Gilbert Wunderlich, Maria Adele Rueger, Michael T. Barbe, Haidar Dafsari, Seda Demir, Sriramya Lapa, Pia S. Zeiner, Adam Strzelczyk, Peter Tinnemann, Christian Kleine, Andreas Totzeck, Stephan Klebe, Agata Mikolajewska, Brigitte G. Dorner, Elisabeth Fertl, Christian Grefkes-Hermann, Gereon Fink, Christoph Kleinschnitz, Tim Hagenacker

**Affiliations:** 1 Department of Neurology and Center for Translational Neuro- and Behavioral Sciences (C-TNBS), University Hospital Essen, University of Duisburg-Essen, Hufelandstr. 55, 45147 Essen, Germany; 2 National Consultant Laboratory for Neurotoxin-producing Clostridia (Botulism, Tetanus), ZBS3, Centre for Biological Threats and Special Pathogens, Robert Koch Institute, Seestr. 10, 13353 Berlin, Germany; 3 Faculty of Medicine, Department of Neurology, University of Cologne, University Hospital Cologne, Kerpener Str. 62, 50937 Cologne, Germany; 4 Department of Neurology, Goethe University Frankfurt, University Hospital, Epilepsy Center Frankfurt Rhine- main, Schleusenweg 2-16, 60590 Frankfurt am Main, Germany; 5 Clinic of Neurology and Neurophysiology, Medical Center, Faculty of Medicine, University of Freiburg, University of Freiburg, Breisacher Str. 64, 79106 Freiburg im Breisgau, Germany; 6 Department of Neurology, Rudolfstiftung C. Landstrasse, Vienna Healthcare Group, Juchgasse 25, Vienna, 1030 Austria; 7 Department of Nephrology and Medical Intensive Care, Charité - Universitätsmedizin Berlin, Corporate member of Freie Universität Berlin and Humboldt Universität zu Berlin, Charitéplatz 1, 10117 Berlin, Germany; 8 Poison Control Center, Charité-Universitätsmedizin Berlin, Hindenburgdamm 30, 12203 Berlin, Germany; 9 Department of Neurology, University Hospital Würzburg, Josef-Schneider-Strasse 11, 97080 Würzburg, Germany; 10 Cognitive Neuroscience, Institute of Neuroscience and Medicine (INM-3), Research Center Jülich, Leo-Brandt- Str, 52425 Jülich, Germany; 11 Department of Neurology, Goethe University Frankfurt, University Hospital, Schleusenweg 2-16, 60590 Frankfurt am Main, Germany; 12 Dr. Senckenberg Institute of Neurooncology, Goethe University Frankfurt, University Hospital, Schleusenweg 2- 16, 60590 Frankfurt am Main, Germany; 13 Competence Center for High Consequence Infectious Diseases (HCID), Gesundheitsamt Frankfurt am Main, Breite Gasse 28, 60313 Frankfurt am Main, Germany; 14 Global Health Sciences Unit, Institute of Social Medicine, Epidemiology and Health Economics, Charité - Universitätsmedizin Berlin, Luisenstraße 57, 10117 Berlin, Germany; 15 Strategy and Incident Response (ZBS7), Centre for Biological Threats and Special Pathogens, Robert Koch Institute, Nordufer 20, 13353 Berlin, Germany

**Keywords:** Iatrogenic botulism, Intensive care, Botulinum neurotoxin, Off-label use

## Abstract

**Background:**

Intragastric botulinum neurotoxin injections (IBNI) are offered off-label in the private medical sector in a few European countries as a safe and effective weight-loss measure. In February and March 2023, an outbreak of iatrogenic botulism occurred in several European countries following IBNI treatment in Turkey. This case series describes the clinical features of severe iatrogenic botulism after IBNI.

**Methods:**

We retrospectively summarize the clinical course and emergency department and intensive care unit interventions in ten cases of severe iatrogenic botulism that occurred after receiving IBNI in this sudden outbreak in Austria and Germany.

**Results:**

Seven out of ten cases initially showed characteristic symptoms of botulism with diplopia, dysphagia, dysarthria, dysarthrophonia, and descending paralysis. All patients were hospitalized, six in an intensive care unit and partially requiring mechanical ventilation. All patients recovered and were discharged without relevant permanent deficits.

**Conclusion:**

Our study highlights ten clinical cases in this iatrogenic botulism outbreak, representing the largest reported outbreak worldwide. Clinicians should be aware of the risks associated with medical procedures involving botulinum neurotoxins and ensure measures to minimize the risk of iatrogenic botulism.

**Supplementary Information:**

The online version contains supplementary material available at 10.1186/s42466-024-00350-3.

## Introduction

Intragastric botulinum neurotoxin injections (IBNI) have been explored as a potential treatment for obesity. The rationale for this application is a delayed gastrointestinal transit, leading to an increase in satiety and decrease in appetite. While small studies have suggested that IBNI may be effective for weight reduction, the evidence remains questionable [23, 28]. In a randomized, controlled trial of 60 obese patients, IBNI did not significantly reduce body weight compared to placebo [25]. A meta-analysis of six randomized, controlled trials, including 192 patients, suggested a significant decrease in weight following IBNI [28]. However, the authors stressed that this effect could only be maintained over time when combined with an appropriate diet and lifestyle change.

Iatrogenic botulism is a rare but potentially severe condition caused by direct exposure to botulinum neurotoxin (BoNT) through medical procedures.

Local injections of BoNT paralyze specific muscles and are approved for therapeutic interventions for selected indications, such as upper and lower limb spasticity following brain injury and dystonia. In addition, BoNT is used successfully worldwide for cosmetic indications, such as the reduction of facial wrinkles. While therapeutic and cosmetic BoNT injections are generally safe, overdosing, improper injection techniques, or using contaminated or counterfeit substances can cause iatrogenic botulism [6, 10, 15, 18]. 

Despite the lack of evidence and approval of regulatory authorities in the USA or EU, IBNI is promoted “off-label” as a safe and effective weight loss measure.

Symptoms of botulism can range from mild to severe, and typically include muscle weakness that can lead to paralysis or respiratory failure. Prompt diagnosis and adequate treatment are essential for a favorable health outcome. Management typically involves early administration of antitoxin, symptomatic therapy with cholinesterase inhibitors, supportive care and, in severe cases, mechanical ventilation [5, 16, 29]. 

We report a series of ten severely affected inpatients suffering from iatrogenic botulism observed and treated in Germany and Austria in early March 2023, following IBNI in Turkey in late February 2023.

## Methods

The first reports of botulism cases were notified to the Robert Koch Institute (RKI), the central institution of the German government for disease surveillance and prevention, in the first week of March 2023 from German or Austrian residents who had returned from Turkey after receiving IBNI for weight reduction there. Within a month, 30 cases were detected in Germany in addition to two cases in Switzerland, and one case each in Austria and in France [9]. To address the situation, a national ad-hoc meeting was convened with the hospitals that had treated patients in intensive care units (ICU). A total of seven patients were treated in ICU at five different hospitals in Germany, and six patients from four hospitals were included in the study. The study also enrolled three less severely affected patients from three other hospitals. The clinical data was collected and reviewed retrospectively by treating physicians in 7 different hospitals in Germany (6) and Austria (1) with the aid of a detailed questionnaire (supplementary data). This questionnaire contained information on symptoms, diagnostic and therapeutic procedures.

Neurotoxin detection in serum was performed at the RKI, applying two newly-developed methods [9] that measure the enzymatic activity of BoNT either by endopeptidase mass spectrometry [27] or by neoepitope-specific monoclonal antibodies [26]. 

## Results

We gathered data from ten patients aged with a median age of 39 (range 22–50) years, eight women and two men, with a median BMI of 28·6 kg/m² (range 27·5–34·4). Symptoms of botulism were apparent a median of 2 (range 2–5) days after IBNI, and patients were admitted to hospital a median of 9 (range 6–14) days after IBNI. In most cases, the first symptoms were blurred vision and slurred speech, followed by muscle weakness and shortness of breath. The worsening of symptoms over several days, dysphagia and dyspnea led to a visit to the emergency room. According to patient self-reports or clinical record, patients were administered abobotulinumtoxinA (BoNT type A, Dysport^®^) at a dose of 1,500 units, with one patient recalling having received 2,500 units. All had IBNI within four days of each other in a private Turkish hospital. No one has ever been injected with BoNT before (see additional Table 1).

The patients developed diplopia (8/10), dysphagia (9/10), dysarthria (9/10), and dysarthrophonia (10/10), as well as descending paralysis (9/10). All patients had dyspnea upon admission (see additional Table 2). Six patients were initially admitted to an intensive care unit due to increasing dyspnea. One patient required temporary noninvasive ventilation two days after admission because of pneumonia, most likely caused by aspiration due to dysphagia. Another patient developed delayed ventilatory failure requiring intubation and subsequent tracheotomy. Swallowing difficulties prompted nasogastric tube feeding in four out of ten cases. With repetitive stimulation, a pathological increase was measured in one case and significantly reduced motor response potentials consistent with botulism were measured in two of five patients who underwent measurements. An initial reduction in vital capacity was observed in all three patients tested. BoNT detection in serum was successful in the endopeptidase mass spectrometry assay in three of six patients tested. Detection was unsuccessful in blood samples collected more than nine days after IBNI. Botulism antitoxin was administered in three out of ten cases. Botulism antitoxin was administered in three out of ten cases nine to ten days after IBNI. Seven patients were treated symptomatically with pyridostigmine, one patient with pyridostigmine and 3,4-diaminopyrimidine. All patients received physiotherapy and speech therapy. The clinical status improved in most cases within a few days, and they were soon released from the hospital. But in some cases, prolonged hospitalization was necessary (median 10.5 (max. 38) days), including a median of 3 (max. 21) days on the intensive care unit. All patients left the hospital with significantly improved symptoms.

## Discussion

We report ten clinical cases of severe iatrogenic botulism requiring hospitalization after IBNI. IBNI, despite any official approval, is offered and promoted via media in the private sector. No cases of botulism had been reported until these patients showed symptoms of botulism after receiving IBNI in the last week of February 2023 in two hospitals in Turkey [9, 12, 14]. As far as we are aware, this is the largest worldwide outbreak of iatrogenic botulism to be reported.

Botulism is a medical emergency necessitating prompt diagnosis and immediate and adequate treatment. Antitoxin therapy is crucial and involves administering botulinum antitoxin to neutralize the toxin. It is most effective when given within 24 h of symptom onset [16, 29], but requires careful medical supervision due to the risk of allergic reactions [20, 29]. However, it cannot reverse existing paralysis.

Symptomatic therapy with cholinesterase inhibitors such as pyridostigmine may alleviate symptoms, even when presynaptic acetylcholine release is blocked by BoNT. This suggests that not all motor units of a muscle have been completely blocked by BoNT, and that unblocked motor units can partially take over the function of the blocked motor units. The 3,4-diaminopyridine used for symptomatic therapy of the presynaptic Lambert Eaton Myasthenic Syndrome may also be used to stimulate presynaptic acetylcholine release and thus attenuate paralysis symptoms. But data are scarce and contradictory [7, 8]. 

Supportive care to prevent aspiration pneumonia and to treat respiratory failure with mechanical ventilation is the essential component of botulism therapy, and is critical for a good clinical outcome [21]. This includes continuous monitoring and repeated testing of respiratory function, speech and motor skills, accompanied by physiotherapy and speech therapy. However, swallowing, speech, and muscle weakness may remain impaired for several months until the presynaptic function blocked by BoNT is completely restored.

Both foodborne and iatrogenic botulism are caused by botulinum neurotoxins. Foodborne botulism involves a broad range of different sero- and subtypes produced by *Clostridium botulinum* in contaminated food, whereas iatrogenic botulism is caused by pharmaceutical preparations of two defined types, BoNT Type A1 and BoNT Type B1. While iatrogenic botulism results from injections of BoNT, and thus follows clear pharmacokinetics, the uptake of BoNT from food is more complex and results in delayed uptake kinetics via the intestinal epithelium into the circulation. In particular, the onset of constipation in foodborne botulism can prolong BoNT uptake. Consequently, unlike iatrogenic botulism, foodborne botulism is often characterized by progredient symptoms. The amount of BoNT in iatrogenic botulism cases is usually extremely low (even after overdosage) because of the low amount of toxin in pharmaceutical preparations. In foodborne botulism, however, BoNT doses range from low to life-threatening amounts resulting in mild symptoms to full body paralysis including respiratory arrest. In both cases, the detection of BoNT from clinical samples is challenging [22], but even more in iatrogenic botulism where minute doses (pg/mL-range and below) have to be detected. As previously reported, BoNT was detected in nine cases out of 12 samples originally tested. Six cases were from our cohort, of which BoNT was detected in three cases [9]. This represents one of the first cases of iatrogenic botulism in which BoNT detection in patient material was successful, despite delayed sampling.

We speculate that intramural depots of BoNT near gastral blood vessels may have resulted in increased uptake into the bloodstream in the ten patients. It cannot be ruled out that prolonged release of BoNT of intramural depots may explain secondary worsening.

Studies on the intragastric injection of BoNT/A to achieve weight loss applied 100–300 units of onabotulinumtoxinA (Botox^®^) [24] or 300–500 units of abobotulinumtoxinA (Dysport^®^) [1, 19]. In our cases, the reported 1500 units of abobotulinumtoxinA seem high, but do not exceed the maximum dose recommended by the manufacturer for medical purposes. Furthermore, it seems plausible that the clinic has used 1500 units for IBNI in the past without experience much adverse effects. There is also a dose-response relationship to be discussed. The one case that received 2500 units was one of the most severely affected and spent the longest time in intensive care.

The most plausible explanation is that much higher doses than prescribed were intentionally or unintentionally administered, since BoNT was detected in the bloodstream more than one week after injection [9]. Higher doses could be due to a manufacturing defect in the batch of abobotulinumtoxinA used. However, this seems very unlikely based on the strict release criteria for pharmaceuticals and the absence of iatrogenic cases occurring elsewhere. Due to massive occurrence of iatrogenic cases within a very limited time window and the majority associated to a single clinic makes an improper administration of IBNI unlikely as no other cases of iatrogenic botulism have been reported elsewhere before or after this outbreak.

A more likely option would be the use of a counterfeit product. Indeed, counterfeit products with a higher-than-stated dose have been observed [13, 17]. It is also worth noting the differences in biological effectiveness between abobotulinumtoxinA (Dysport ^®^) and e.g. onabotulinumtoxinA (Botox ^®^). Due to differences in the composition of the toxin and excipients abobotulinumtoxinA has to be applied in higher units to reach a comparable effect as onabotulinumtoxinA [2–4] even though the quantity of BoNT/A1 is comparable [11]. This is also reflected in the manufactures’ maximum recommended doses for medical purposes of 1500 units for abobotulinumtoxinA and 400 units for onabotulinumtoxinA. Exemplarily, a counterfeit onabotulinumtoxinA of 1,500 units which reaches the market mislabeled as a fake 1,500 units of abobotulinumtoxinA would result in an overdose due to the up to 5-fold higher biological potency of onabotulinumtoxinA.

More likely, however, is the use of overdosed counterfeit product or counterfeit product mislabeled with another BoNT/A and indeed, counterfeit products have been associated with an outbreak of iatrogenic botulism in the past [18].

## Conclusion

We presented a medical problem known for a considerable time involving one of the most lethal toxins worldwide, manifesting in an unfamiliar and concerning manner. Clinicians must be aware of the risks associated with medical procedures involving botulinum neurotoxins, especially unapproved “off-label” use, and take appropriate measures to minimize the risk of iatrogenic botulism. Every neurologist and emergency physician should be aware of botulism. Although it is rare, it is a life-threatening condition that requires prompt action, as antitoxin administration is only effective in the early stages. Full recovery is expected if patients are treated appropriately. As off-label use of BoNT may not always be reported, patients presenting with symptoms of botulism should be actively asked about off-label use of BoNT.

## Electronic supplementary material

Below is the link to the electronic supplementary material.

Supplementary Material 1

## Data Availability

The data are available from the corresponding author and can be requested.

## Abbreviations

BoNT: Botulinum Neurotoxin.
IBNI: Intragastric botulinum neurotoxin injection.
ICU: Intensive care units.
RKI: Robert Koch Insitute.
